# Age-friendly neighbourhoods and physical activity of older Surinamese individuals in Rotterdam, the Netherlands

**DOI:** 10.1371/journal.pone.0261998

**Published:** 2022-01-27

**Authors:** Warsha Jagroep, Jane M. Cramm, Semiha Denktaș, Anna P. Nieboer

**Affiliations:** 1 Department of Socio-Medical Sciences, Erasmus School of Health Policy & Management, Erasmus University Rotterdam, Rotterdam, the Netherlands; 2 Department of Psychology, Education and Child Studies, Erasmus School of Social and Behavioural Sciences, Erasmus University Rotterdam, Rotterdam, the Netherlands; PLOS: Public Library of Science, UNITED KINGDOM

## Abstract

**Background:**

Age-friendly neighbourhoods seem to promote physical activity among older individuals. Physical activity is especially important for chronically ill individuals. In the Netherlands, older Surinamese individuals are more likely to have chronic diseases than are their native Dutch counterparts. This study examined relationships of neighbourhood characteristics with physical activity among older Surinamese individuals in Rotterdam, the Netherlands.

**Methods:**

Of 2749 potential participants, 697 (25%) community-dwelling older (age ≥ 70 years) Surinamese individuals living in Rotterdam, the Netherlands, completed a questionnaire on personal and neighbourhood characteristics between March and June 2020. Correlation and multilevel regression analyses were performed to identify associations between missing neighbourhood characteristics for ageing in place and physical activity.

**Results:**

Scores for the neighbourhood domains communication and information (*r* = -0.099, *p* ≤ 0.05), community support and health services (*r* = -0.139, *p* ≤ 0.001), and respect and social inclusion (*r* = -0.141, *p* ≤ 0.001), correlated negatively with participants’ PA. In the multilevel analysis, overall missing neighbourhood characteristics to age in place scores were associated negatively with physical activity (*p* ≤ 0.05).

**Conclusion:**

This study showed the importance of age-friendly neighbourhoods for physical activity among older Surinamese individuals in Rotterdam, the Netherlands. Our findings suggest that the neighbourhood plays an important role in supporting older individuals’ leading of physically active lifestyles. Further research is needed to support the development of interventions to create age-friendly neighbourhoods.

## Introduction

The health of older Surinamese individuals in the Netherlands is worse than that of the native Dutch population. These individuals are more likely to have (multiple) chronic diseases (e.g. hypertension, type 2 diabetes mellitus) [1–5] and to experience psychological distress [6] than are native Dutch individuals. Additionally, older Surinamese individuals have a greater risk of death from these chronic diseases than do their native counterparts with the same socio-economic backgrounds or educational levels [1–4]. Physical activity (PA) plays a significant role in the prevention of many chronic diseases, including hypertension and type 2 diabetes [7–10]. PA involves all movements including actively commuting (walking, cycling), household activities, and leisure-time activities (sports, walking, gardening, cycling) [11]. Despite current knowledge about the importance of PA, a decline in the average activity level with age [12, 13] and a low PA level among Surinamese individuals in the Netherlands [14] have been observed.

Neighbourhood characteristics provide potential opportunities and barriers to engagement in physically active lifestyles [15]. It becomes more important as people age, likely because older individuals spend more time in their neighbourhoods than do their younger counterparts [16–18]. This makes them more dependent on the social and physical infrastructure of the neighbourhood. Thus, the investigation of associations between neighbourhood characteristics and PA among older individuals is of particular relevance. In addition, declining physical and mental health and increased fragility reduce older individuals’ ability to cope with environmental demands [19–21], and these qualities are more prevalent among older Surinamese individuals than among older individuals born in the Netherlands [22, 23].

Rotterdam is the second largest city in the Netherlands, and it hosts 19% of older individuals who migrated from non-Western countries, of which Surinamese individuals form the largest group [24]. On average, Rotterdam has a lower socio-economic status than the Netherlands in general [25]. Health deprivation and worse perceived health are also more prevalent in this city than in other Dutch cities [26]. Individuals who migrated from non-Western countries are concentrated highly in large cities and often live in deprived neighbourhoods [27, 28], which may entail low levels of greenness [29], poor services provision and a lack of social cohesion [30], in turn impairing physically active lifestyles.

The World Health Organization (WHO) identified eight domains for the description of neighbourhood characteristics in ‘age-friendly’ cities [31]: 1) outdoor spaces and buildings, 2) housing, 3) transportation, 4) communication and information, 5) community support and health services, 6) respect and social inclusion, 7) social participation, and 8) civic participation and employment. These domains are likely to be associated with the PA levels of older individuals and are discussed individually below [32].

### Research aim

Age-friendly neighbourhood characteristics seem to be associated with the PA of older individuals. However, the current literature lacks studies of the effects of neighbourhood characteristics on diverse societal subgroups [33]. As older Surinamese individuals are more likely to develop chronic conditions, compared to native Dutch individuals, that can be prevented by regular PA, the aim of this study was to examine associations of neighbourhood characteristics with PA levels among older Surinamese individuals in Rotterdam, the Netherlands. We hypothesised that physical activity would be associated negatively with missing neighbourhood characteristics among older Surinamese individuals.

### Outdoor spaces and buildings

Older individuals have emphasised the importance of walkability (e.g. the presence of walking surfaces, sidewalks, bike lanes) for the performance of PA in their neighbourhoods [34–36]. Neighbourhood infrastructure seems to be important for the improvement and/or maintenance of individuals’ PA levels. For example, greater street connectivity [37, 38], (perceived) traffic safety [39, 40], distances to destinations such as parks and stores [38, 41, 42] and access to these destinations [35] have been associated with more walking and bicycling. These factors might be especially important for older individuals due to, for example, mobility limitations resulting in the use of a walker or wheelchair. Terrain features such as steps and uneven surfaces [43] might be barriers for neighbourhood PA. Conversely, traffic lights [44], street lighting [44–47] and facilities such as benches and toilets are important facilitators [45, 48]. For example, the timing of traffic lights at pedestrian crossings must consider the walking speed of crossing users, and research has indicated that older individuals become delayed as the traffic volume increases [49]. Furthermore, attractive and green open spaces lead to more PA [36, 38].

### Housing

Indoor aspects also play important roles in the activity levels of older individuals. Suitable housing is an important facilitator of older individuals’ PA [50]. For example, wide doors and non-slippery floors enable older individuals, including those who use walkers and wheelchairs, to move about in their homes. Even floors make it easier for older individuals to go outside (e.g. for grocery shopping or a walk) and to be physically active. The availability of home modification programmes might also be essential for older individuals without limitations. For example, research, has indicated that older individuals with home modifications (e.g. railings, bathroom modifications) are less likely to experience declines in physical functioning and falls [51]. In addition, home modification has been shown to improve the activities of daily living of older individuals with and without limitations [52, 53], making them less dependent on others and more likely to be active on their own.

### Transportation

Transportation involves movement from place to place, for example by car, public transportation, walking or cycling. Regardless of cultural and policy differences, the car is the most commonly used mode of transport by older populations worldwide [54–56]. Various health conditions associated with ageing (e.g. visual impairment, dementia, Parkinson’s disease) may negatively impact driving performance [57–59]. However, the largest proportion of older drivers is considered to be healthy [60]. Driving-related facilities might facilitate PA among older individuals. For example, the presence of parking lots (e.g. at shopping malls or parks) has been associated positively with walking in this population [61], and thus might increase PA.

Access to public transportation (including stops and vehicle features such as priority seating, low steps and non-slippery floors) might be another significant contributor to PA among older individuals [62, 63]. It seems to be associated with older individuals’ walking in their neighbourhoods [34, 64]. Older individuals have emphasised the importance of the proximity of public transportation stops [65], stops at key destinations (e.g. health care and shopping centres) and well-connected routes [31] to their use of public transportation. The availability of information such as clear time tables, routes and signage in public vehicles also seems to be an important factor [31].

Walking and cycling are common forms of neighbourhood transportation. The distance to a given destination seems to be an important contributor to individuals’ decision to walk or cycle [66, 67]. Neighbourhoods with greater residential density, mixed land use and grid-like street patterns with short blocks have been shown to enhance the use of walking and cycling for transportation [68]. In addition, perceptions about traffic and busy roads seem to be associated with walking for a particular purpose [35].

### Communication and information

Informing older individuals about neighbourhood services and programmes is important to make them aware of these opportunities [69, 70], and might increase their participation, for example, in PA programmes [71]. However, the location and source of information provision must be considered. For example, older individuals appreciate the availability of information at locations that they frequent; information provision by individuals in close, regular contact with older individuals, such as health care providers, is also essential [72–74]. Older individuals have emphasised the importance of knowing where to look for information [75]. In addition, the format and design of materials (e.g. use of large font and understandable language, sound quality) are important to make information accessible for older individuals [31]; appropriate designs contribute to their healthy behaviours, including PA [76].

### Community support and health services

Community and health services provide formal support, such as in general practitioner (GP) practices and pharmacies, and informal support, such as that of neighbours and volunteers. These services are vital to the maintenance of older individuals’ health and independence [31], and eventually might have impacts on their PA, which takes place most often in community settings. Neighbours can be important facilitators of PA among older individuals [47], who prefer and respond best to face-to-face social support and peer coaching [77–80]. Such individualised support seems to engage older individuals in PA, as it provides them with advice and information from non-professionals with common backgrounds (e.g. similar life experiences, shared characteristics) who help them to reach shared goals [78, 80, 81]. Thus, the provision of PA sessions that involve face-to-face social support and/or peer coaching in the community might encourage older individuals to be physically active.

Research suggests that GPs can effectively promote PA with simple positive-reinforcement messages and the provision of specific plans for fitness-related activities, known as ‘PA prescriptions’ [82, 83]. In addition, GPs seem to play an important role in the provision of information (e.g. about community PA programmes and groups) to older and chronically ill individuals, thereby facilitating participation in neighbourhood activities [84].

### Respect and social inclusion

The attitudes, behaviour and messages of individuals in the community toward older individuals should convey respect and social inclusion. The degree to which this is true affects the range of opportunities offered to older individuals for social participation, entertainment and/or employment. Greater neighbourhood social cohesion has been shown to facilitate older individuals’ participation in community-based activities overall, and specifically to increase their engagement in PA [85–89]. In addition, a sense of belonging has been found to be important for older individuals’ participation in activities [90]. Community belonging is associated with healthy behaviours such as walking [91], and it encourages networking [92]. Finally, (perceived) neighbourhood safety and fear of violence influence individuals’ activity levels [39, 93].

### Social participation

Participation creates opportunities for older individuals to be physically active and to broaden their networks [94]. Conversely, older individuals with limited social participation are less likely to be physically active [95]. Thus, the creation of opportunities for older individuals to participate socially and to create networks might eventually increase their PA.

### Civic participation and employment

Civic participation and employment (paid or unpaid), such as (flexible) job opportunities, job training, volunteer work and involvement in decision-making bodies, provide opportunities for older individuals to exercise citizenship. Older individuals who volunteer might also be more physically active [96]. However, volunteer opportunities need to be accessible and tailored to older individuals’ capabilities and interests [31, 97]; visual impairment, for example, has been found to reduce the community participation of older individuals [98]. The promotion of volunteer work, which has been found to be a successful predictor of older individuals’ social connectedness [99], might eventually lead to older individuals’ engagement in PA.

## Methods

### Population

In the Netherlands, Surinamese individuals form one of the largest groups with non- Western migration backgrounds. Surinam is a former Dutch colony that obtained independence in 1975. Surinamese individuals migrated to the Netherlands in two main waves, seeking higher education and work and due to political unrest, respectively [100]. The population of Surinam is heterogeneous in terms of culture and geographic origin, including Javanese, Surinamese Chinese, Surinamese Creole (of West African descent), and Surinamese Hindustani (of Indian descent) groups [101]; the population in the Netherlands is comprised mainly of individuals with the latter two backgrounds. Individuals with comparable Surinamese Creole and Surinamese Hindustani backgrounds can also be found in other European countries, such as United Kingdom. In general, most Surinamese individuals speak Dutch well, as Dutch is an official language in Surinam and is used in education, government and the media; this characteristic distinguishes this group from other older individuals who migrated to the Netherlands with limited Dutch language proficiency. Community-dwelling Surinamese individuals aged 70 years and older and living in Rotterdam, the Netherlands, participated in this research.

### Recruitment and questionnaire administration

Potential participants were identified using Rotterdam’s municipal register and asked to participate in this study between March and June 2020. In total 2749 potential participants were contacted, nested in 55 neighbourhoods. Neighbourhoods were classified using four-digit postal codes assigned by the Dutch government. Questionnaires and self-addressed envelopes were distributed via post, followed by a postal reminder. An informational leaflet explaining the aim of the study and its anonymous and voluntary nature was provided to the respondents. Informed consent (written) was obtained from all participants. The first author’s contact information was provided in case potential participants had additional questions. No (financial) incentive was provided. The Ethics Review Committee of Erasmus University Rotterdam approved this study (application no. 19–048) and determined that the rules imposed by the Dutch Medical Research Involving Human Subjects Act did not apply.

Of 2749 older Surinamese individuals contacted, 34 were found to be ineligible due to medical conditions (e.g. dementia, rehabilitation), nursing home residence, change of address or death. Thus, the number of eligible participants was 2715. Of them, 697 individuals filled in the questionnaire (25% response rate).

### Measures

#### Missing neighbourhood characteristics to age in place

Neighbourhood characteristics were evaluated using an instrument developed and utilised in previous research [102–104], applying the WHO framework for age-friendly cities (2007) and additional research [31, 105] (S1 Appendix).

As the questionnaire was developed among the general population of older individuals in the Netherlands [102], we assessed its suitability for the older Surinamese population with four 70-year-old Surinamese individuals in the Netherlands in December 2019–January 2020. As a result, we added two items to the questionnaire; ‘A neighbourhood where individuals help me, for example with a chore or to bring me somewhere’ (community support and health services) and ‘A neighbourhood where social activities are organized specially for Surinamese older individuals’ (social participation). Participants were asked to indicate whether they missed neighbourhood components using a five-point scale ranging from 0 (‘not at all’) to 4 (‘extremely’). Twenty-six items representing the eight age-friendly city domains recognized by the WHO were assessed. Examples by domain are ‘Public buildings with elevators that are easily accessible for wheelchairs and walkers’ (outdoor spaces and buildings, 4 items), ‘Suitable housing for older individuals’ (housing, 2 items); ‘Good public transport’ (transportation, 2 items), ‘Local newspaper with information about what’s going on in the neighbourhood’ (communication and information, 2 items), ‘A neighbourhood with the GP and pharmacy at walking distance’ (community support and health services, 6 items), ‘A neighbourhood where individuals have respect for older individuals’ (respect and social inclusion, 5 items), ‘Affordable activities for older individuals’ (social participation, 3 items) and ‘A neighbourhood where older individuals are involved, for example concerning changes in the neighbourhood’ (civic participation and employment, 2 items). Mean total and domain scores were calculated; higher scores represented more missed neighbourhood characteristics. The Cronbach’s alpha value for the mean total was 0.894, indicating excellent reliability. Cronbach’s alpha value for the subscales ranged from 0.531 to 0.869. We also checked if deleting items resulted in a better Cronbach’s alpha, which was only the case for one item (‘A neighbourhood with people of the same ethnic background as me’). Previous research, however, showed that this item was important for the general older Dutch population [102, 103]. Therefore, we decided to keep this item which allowed us to examine the importance of this item among older Surinamese people.

#### Physical activity

PA was assessed by asking respondents on how many days per week they were physically active for at least 30 minutes. The questionnaire items covered active commuting (walking, cycling), household activities and leisure-time activities (sports, walking, gardening, cycling). We assessed physical activity by asking participants on how many days per week they were physically active. This question is from the validated and reliable short questionnaire to assess health-enhancing physical activity (SQUASH) [106, 107]. Government agencies use this instrument to monitor the PA of the Dutch population [108]. Scores ranged from 0 (not physically active for 30 minutes on any day during the week) to 7 (physically active every day of the week). PA scores were dichotomised as meeting (1; 30 minutes PA per day on at least 5 days of the week) and not meeting (0) the Dutch standard for healthy physical activity [109].

#### Number of chronic diseases

A questionnaire was used to identify the presence of chronic diseases and inquired the following question: “Have you had any of the following diseases or conditions in the previous 12 months?” (0 = no, 1 = yes). A list of 10 chronic conditions (i.e. cardiovascular diseases, diabetes, lung diseases) adopted from O’Halloran et al. [109] was provided. Participants were also asked to report unlisted conditions, which resulted in the reporting of 51 additional conditions, including kidney failure and limited vision. These conditions (denoted ‘other chronic diseases’) were taken into account when we counted chronic diseases. We allocated participant-reported conditions already listed on the questionnaire to the appropriate listed options. Participants also reported risk factors for chronic diseases, such as high cholesterol and high blood pressure, which we did not include in the analysis. Simple count was used in the analyses.

#### Socio-demographic variables

The questionnaire solicited information on respondents’ age, gender (male or female) and marital status (living alone/widowed/divorced or married/living with a partner). Respondents were asked to report the highest educational level completed in the Netherlands or abroad, with the option to write unlisted forms of schooling. This variable was dichotomised as low (completion of elementary school or less) and high (more than elementary school). Income levels were determined based on respondents’ reported monthly household incomes, including social benefits, pensions and alimony. Response options ranged from ‘less than €1000 a month’ (1) to ‘€3050 or more a month’ (4), with a fifth ‘do not know/ do not want to tell’ option provided. This variable was dichotomised as low (less than €1350 a month) and high (€1350 or more a month).

### Statistical analyses

The SPSS software (version 26; IBM Corporation, Armonk, NY, USA) was used to analyse the data. Descriptive statistics (means, minimums, maximums, standard deviations and/or percentages) were calculated for all variables. Assessment for multicollinearity yielded tolerance values > 0.3 and variance inflation factors < 3, indicating no sign of multicollinearity. Spearman correlation analysis was performed to identify associations of background characteristics and missed neighbourhood characteristics with PA. Two-sided *p* values ≤ 0.05 were considered to be significant. We found that the neighbourhood level significantly affected PA by comparing the –2 log likelihoods of the regression models containing PA only and containing PA and the neighbourhood level (S2 Appendix). We thus employed multilevel regression analyses to account for the clustering of our data; older Surinamese individuals (level 1) were nested in 55 neighbourhoods (level 2). Unaggregated individual data were used for all analyses. In the multilevel model, besides a random intercept on neighbourhood level, we evaluated the necessity of adding random slopes for the different covariates (age, sex, marital status, education, income, and number of chronic diseases). This evaluation was performed with likelihood ratio tests. Furthermore, because age is the only covariate with a non-meaningful zero, the centred value of age was used in the multilevel modelling. All multilevel analyses were performed with the mixed procedure in STATA (version 17) using maximum likelihood. The regression coefficients in the mixed procedure were tested with the z-test.

## Results

Table 1 displays the characteristics of the 697 study participants; 54.2% were women, 67.4% were unpartnered, 38.5% had low educational levels and 49.6% had low incomes. The mean age was 76.2 ± 4.9 (range 70–100) years and the mean number of chronic diseases was 1.6 ± 1.4 (range 0–8). The missing neighbourhood characteristic scores ranged from 0.90 ± 0.90 to 1.4 ± 1.3 (range 0–4), suggesting that the respondents found their neighbourhoods to be moderately to highly age friendly. On average participants were physically active on 3.7 days per week; 39.8% of the participants met the PA standard.

**Table 1:** Descriptive statistics for the study population (*n* = 697).

Characteristic	Range	% or mean (SD)
Gender (female)^a^		54.2
Age	70–100	76.2 (4.9)
Marital status (unpartnered)^b^		67.4
Education (low)^c^		38.5
Income (low)^d^		49.7
Number of chronic diseases	0–8	1.6 (1.5)
Missing neighbourhood characteristics to age in place		
Outdoor spaces and buildings	0–4	1.1 (1.0)
Housing	0–4	1.4 (1.3)
Transportation	0–4	1.1 (1.1)
Communication and information	0–4	1.0 (1.0)
Community support and health services	0–4	1.0 (1.0)
Respect and social inclusion	0–4	1.1 (0.9)
Social participation	0–4	1.4 (1.2)
Civic participation and employment	0–4	0.9 (0.9)
Overall missing neighbourhood characteristics to age in place	0–4	1.1 (0.8)
Number of days physically active	0–7	3.7 (2.4)
Meeting physical activity standard		39.8

SD, standard deviation.

^a^ = reference category is male,

^b^ = reference category is partner,

^c^ = reference category is high education,

^d^ = reference category is high income.

Table 2 displays the results of the correlation analyses. Age (*p* ≤ 0.01), unpartnered status (*p* ≤ 0.01), low educational level (*p* ≤ 0.01), low income (*p* ≤ 0.001), and number of chronic diseases (*p* ≤ 0.001) were associated negatively with PA. In addition, we found negative correlations of PA with the domains communication and information, community support and health services, respect and social inclusion and overall missing neighbourhood characteristics to age in place scores (*r* = -0.099 to -0.141, all *p* < 0.05).

**Table 2:** Correlations of participant and missing neighbourhood characteristics to age in place with physical activity (n = 697).

Variable	Physical activity		
	n	r	*p*
Age (years)	633	-0.123	0.002**
Gender (female)^a^	633	0.038	0.339
Marital status (unpartnered)^b^	618	-0.114	0.005**
Education (low)^c^	617	-0.107	0.008**
Income (low)^d^	596	-0.148	<0.001***
Number of chronic diseases	621	-0.153	<0.001***
Missing neighbourhood characteristics to age in place scores			
Outdoor spaces and buildings	601	-0.084	0.038
Housing	609	-0.020	0.622
Transportation	612	-0.076	0.060^#^
Communication and information	585	-0.099	0.016*
Community support and health services	588	-0.139	0.001***
Respect and social inclusion	592	-0.141	<0.001***
Social participation	610	-0.003	0.947
Civic participation and employment	586	-0.072	0.082^#^
Overall missing neighbourhood characteristics to age in place^a^	558	-0.114	0.007**

n = sample size, r = correlation coefficient.

^a^ = reference category is male,

^b^ = reference category is partner,

^c^ = reference category is high education

^d^ = reference category is high income.

^#^*p* ≤ 0.10,

*p ≤ 0.05,

**p ≤ 0.01,

***p ≤ 0.001.

The results of the multilevel analyses are presented in Table 3. Regarding the random slopes, we found that a random slope for education significantly improved the model. Adding random slopes for the other covariates did not significantly improve the model. So, the final multilevel model contained a random intercept and a random slope for education (see appendix for the final multilevel model; S2 Appendix).

**Table 3:** Associations of participant and neighbourhood characteristics to age in place with physical activity, as determined by multilevel analysis.

Variable	Physical activity		
	B	95% CI	*p* *
Constant	4.63	4.17 to 5.08	<0.001
Age (years; centred)	-0.10	-0.14 to -0.06	<0.001
Gender (female)	0.36	0.05 to 0.78	0.09
Marital status (unpartnered)	-0.54	-1.0 to -0.08	0.02
Education (low)	-0.44	-0.99 to 0.11	0.11
Income (low)	-0.13	-0.56 to 0.30	0.56
Number of chronic diseases	-0.38	-0.78 to 0.01	0.06
Overall missing neighbourhood characteristics to age in place	-0.34	-0.58 to -0.10	0.005

B = unstandardized regression coefficient derived from the mixed procedure in STATA, CI = Confidence Interval.

*p values based on the z-test.

After controlling for background characteristics age (*p* < 0.01) and unpartnered status (*p* = 0.02) were significantly associated negatively with PA (Table 3). In addition, the overall missing neighbourhood characteristics to age in place score was associated negatively with PA (*p* = 0.005). Unlike the correlation analysis, the multilevel regression analysis revealed no significant association of PA with low educational level (*p* = 0.11), low income level (*p* = 0.56) or number of chronic diseases level (*p* = 0.06).

## Discussion

This study demonstrated the importance of neighbourhood characteristics for older Surinamese individuals’ PA. On average, participants considered their neighbourhoods to be moderately to highly age friendly, perhaps because Rotterdam has implemented programmes to develop supportive neighbourhoods for older individuals (e.g. Let’s Talk [*Even Buurten*], NEW ROADS) for decades [110, 111].

Although similar findings have been obtained for the general older population [32, 112], this study is the first to show associations between neighbourhood characteristics and PA among older Surinamese individuals, although the effect sizes were small. As human behaviour responds to neighbourhood characteristics, facilitative changes in the environment are likely to support PA and improve subsequent health outcomes. The crucial role of local governments in providing age-friendly neighbourhoods is acknowledged [105, 113]. Our findings have implications for policy makers and service providers aiming to build and maintain age-friendly communities that support older individuals’ PA, in turn benefitting health and potentially reducing care costs [114, 115].

In particular, this study showed a weak association between *outdoor spaces and buildings* and PA levels among older Surinamese individuals. In line with previous literature, our research suggests that attention should be given to features such as walkability (e.g. sidewalks, safe crosswalks), infrastructure (e.g. greater street connectivity, traffic safety) and the provision of attractive and green open spaces [34, 64, 116, 117]. Walkability has been found to promote walking as a form of transportation [118]. Thus, the presence of nearby destinations (e.g. grocery stores) in walkable neighbourhoods might increase PA among older individuals. In addition, public building accessibility seems to be essential for PA. The involvement of various actors (e.g. architects, contractors, customers) in designing public spaces is important to achieve optimal accessibility [119].

The *communication and information* domain was also correlated with PA among older Surinamese individuals. Native and non-native older individuals have emphasised the importance of knowing where to look for information, which is not always easy [75]. Older individuals, for example, often struggle with finding information about neighbourhood activities and social- and health-related matters. Previous research has indicated that health services contexts (e.g. health care sites and health-related events) are essential places at which such information is provided [120]. However, the consideration of channels that reach older migrants, such as local newspapers and the post, is also important [120]. Older migrants prefer to receive information via printed materials or directly from other people [121, 122]. Efforts to promote access to information, including the implementation of effective communication systems that reach migrant older individuals and a focus on accessible (oral and printed) forms of communication, seem to be essential for the promotion of PA [123].

Our findings support that the domain *community support and health services* is associated with PA among older Surinamese individuals. The social element of activities is an important motivator of native and non-native older individuals’ participation in PA and maintenance of physically active lifestyles [80, 123–127]. Older individuals have emphasised that making new friends, socialising and encouraging other participants during group activities motivate them to be physically active [124]. The provision of social support in the community setting has been shown to effectively increase PA [128]. The creation of environments that facilitate social interaction and the formation of new friendships, which eventually become sources of social support, may motivate older individuals to engage in PA [129–131]. For example, the sharing of food and drink after a PA session creates an opportunity to socialise in addition to being physically active. Such approaches may be especially important for older migrants, who sometimes lack PA-related social support [131]. Thus, community-based PA promotion may enhance PA levels among older Surinamese individuals.

In line with our findings, previous research has demonstrated the potential of PA promotion via neighbourhood health services [132–137]. PA counselling programmes implemented through primary health care, for example, have been shown to be feasible and cost-effective strategies for the promotion of PA [132–135, 138]. Thus, health care professionals’ guidance, such as the provision of verbal advice about PA or the mailing of pamphlets on exercise, is important to encourage PA among older Surinamese individuals [136].

Next, the domain *respect and social inclusion* was correlated with PA among older Surinamese individuals. The feeling that one is respected and socially included is known to be related to PA among community-dwelling older individuals [137, 139]. Accordingly, persistent disrespectful attitudes and ageism have been recognised as important barriers to the development of effective public healthy-ageing policies [140, 141]. Therefore, the ways in which ageing and older individuals are represented should be considered during the development of health interventions.

These findings are particularly relevant for policy makers, as they aid the targeting of neighbourhood characteristics for the development of supportive environments that encourage PA among older individuals. Neighbourhood interventions that promote PA among older individuals have been shown to yield significant results in improving health [142]. Although the WHO framework for age-friendly cities was developed for the older population, it might also benefit the general population [143]. Other factors (e.g. gender, income level, ethnicity, health status) also must be considered when developing interventions to promote PA, as these factors have been associated with behaviour changes and PA levels [144]. In addition, individuals’ capabilities should be considered, as neighbourhood characteristics have stronger effects on the behaviour of individuals with than of those without functional limitations [145].

We did not find a significant relationship between the domains *social participation* and *civic participation and employment* with PA among Surinamese older individuals. A study conducted with individuals aged ≥ 55 years in Ireland showed that community participation was related to a greater frequency of meeting friends socially with PA [32]. However, research on the relationship between social participation and PA among older migrants remains scare [146]. For older Surinamese individuals, a supportive environment (as reflected by community support and health services, as well as respect and social inclusion) seems to be more important than actual participation in given social activities for PA engagement. Given the age of our population (70 years and older), civic participation and employment are expected to be less relevant to PA.

Although previous research has indicated that housing has been associated with PA among older individuals, our study did no find an association between *housing* and PA among Surinamese older individuals [147]. Older individuals move mainly to better-quality housing in the same neighbourhood [148, 149], maintaining the advantage of a supportive home without the loss of social connections, outdoor routines and emotional bonding to a familiar place [150]. Participants in this study may not have been thinking about their possible future housing needs, or whether their neighbourhoods accommodated them (or whether they would have to move farther away to meet those needs). Another explanation might be that from an international perspective, homes in the Netherlands are of high quality [151]. More specifically, Rotterdam provides residents with many opportunities for home modification, such as the installation of grab bars, rails and raised toilet seats, resulting in fewer home hazards and improvement in activities of daily living (e.g. dressing) and instrumental activities of daily living (e.g. preparing meals, housekeeping activities) [152, 153]. Thus, study participants’ homes may have already supported their capabilities, explaining the lack of association with PA.

Finally, we found no association between the domain *transportation* and PA. In Rotterdam, older individuals (age ≥ 65 years) are qualified to use public transportation for free. In addition, neighbourhood buses are available for individuals aged ≥ 55 years and those with disabilities. For individuals aged 75 years, the municipality of Rotterdam provides an opportunity to travel by bus after 7 pm under supervision. Individuals with disabilities (condition duration ≥ 6 months, no full recovery possible, not able to walk more than 100 meters without a break) are able to request a European parking card, which permits them to park for free in spots reserved for disabled individuals (marked with a wheelchair symbol) at busy destinations, such as shopping malls and health care centres. The card also qualifies holders for a private parking space on their license plates to park near their home. The existence of these transportation privileges and facilities may explain the lack of association with PA. In neighbourhoods without such facilities for older individuals, findings are likely to differ.

This study has several limitations that should be considered when interpreting our findings. Given that the data collection took place during the onset of the COVID-19 pandemic, a separate analysis was performed to determine whether PA levels differed between participants who filled in the questionnaire before and after COVID-19 measures (S3 Appendix) proposed by the National Institute for Public Health and the Environment which were taken in the Netherlands (S4 Appendix). Reported PA levels were low initially and did not decrease after the introduction of the COVID-19 measures. Next, given the heterogeneity of the Surinamese population in the Netherlands, additional analyses were performed to determine whether ethnicity (Javanese, Surinamese Chinese, Surinamese Creole and Surinamese Hindustani) significantly affected PA (S5 Appendix). No significant difference in PA was found among ethnic groups. Additionally, the cross-sectional design of this study prevented us from determining the causality of relationships. However, our results showed significant association between neighbourhood characteristics and PA, which is an essential step that prompts further studies to analyze directionality. A longitudinal study design is needed to investigate the relationship between ageing in place and PA over time. Furthermore, this study was conducted in Rotterdam, the Netherlands; research in other regions is needed to understand differences among municipalities and their effects on older individuals’ PA. Although some facilities and privileges (e.g. European parking card) are regulated nationally, neighbourhood characteristics such as community support, respect and social inclusion are expected to show regional differences. Thus, future research should involve comparison among municipalities and/or countries. Finally, the effect sizes in this study were small, indicating that the relationships between neighbourhood characteristics and PA were weak [154].

## Conclusion

This study showed that the absence of neighbourhood characteristics for ageing in place is associated negatively with PA among older Surinamese individuals in Rotterdam. The findings represent a first step toward the development of interventions and policies contributing to the age-friendliness of neighbourhoods for these older individuals, including support of PA. A longitudinal follow-up study conducted in a variety of settings is needed to examine potential causal pathways and to identify differences among municipalities and their effects on older individuals’ PA over time.

## Supporting information

S1 Appendix Instrument to assess missing neighbourhood characteristics to age in place. (DOCX)

S2 Appendix Multilevel model on missing neighbourhood characteristics for ageing in place and physical activity. (DOCX)

S3 Appendix Physical activity before and after implementation of COVID-19 measures. (DOCX)

S4 Appendix COVID-19 measures taken in the Netherlands. (DOCX)

S5 Appendix Physical activity of Creole, Hindustani and Other Surinamese. (DOCX)

S6 Appendix Dataset—Physical activity among older Surinamese adults (SPSS). (SAV)

S7 Appendix Dataset—Physical activity among older Surinamese adults (STATA). (DTA)
