# Supplementary material for: Age-friendly neighbourhoods and physical activity of older Surinamese individuals in Rotterdam, the Netherlands
Source: PLoS One. 2022 Jan 27;17(1):e0261998. doi: 10.1371/journal.pone.0261998 (PMC8794150; doi:10.1371/journal.pone.0261998)
Supplement: S1 Appendix — (DOCX) [file pone.0261998.s001.docx]

**S1 Appendix. Instrument to Assess Missing Neighbourhood Characteristics to Age in Place**

Developed by van Dijk and colleagues. Published in final edited form in: Ageing and Society, 2015;35(8):1771.

We would like to know what you are missing in your neighbourhood in order to be able to live there as long as possible. Please choose one of the following answer options: Not at all, Slightly, Quite, Very, Extremely.

*Outdoor spaces and buildings*
A clean and green neighbourhood.
A neighbourhood with wide sidewalks and safe crosswalks.
Public buildings with elevators that are easily accessible for wheelchairs and walkers.
A safe neighbourhood.

*Housing*
Affordable housing.
Suitable housing for older people.

*Transportation*
Good public transport.
Sufficient parking spots.

*Communication and information*
Local newspaper with information about what’s going on in the neighbourhood.
Access to internet and internet courses in the neighbourhood.

*Community support and health service*
A neighbourhood where people help me, for example with a chore or to bring me somewhere.
A neighbourhood where home care is easily accessible.
A neighbourhood with the GP and pharmacy at walking distance.
A neighbourhood with places where older people can go for advice and support.
A neighbourhood with volunteers who provide help when necessary.
A neighbourhood with shops and other facilities within walking distance.

*Respect and social inclusion*
A neighbourhood where people have respect for older people.
A neighbourhood where people are willing to help each other whenever necessary.
A neighbourhood with people having the same ethnical background as me.
A neighbourhood where people dare to speak up to each other.
A neighbourhood where people great and talk to each other.

*Social participation*
A neighbourhood where many social activities are organized.
A neighbourhood where social activities are organized specially for Surinamese older people.
Affordable activities for older people.

*Civic participation and employment*
A neighbourhood with possibilities for voluntary work.
A neighbourhood where older people are involved, for example concerning changes in the neighbourhood.
