# Supplementary material for: Age-friendly neighbourhoods and physical activity of older Surinamese individuals in Rotterdam, the Netherlands
Source: PLoS One. 2022 Jan 27;17(1):e0261998. doi: 10.1371/journal.pone.0261998 (PMC8794150; doi:10.1371/journal.pone.0261998)
Supplement: S2 Appendix — (DOCX) [file pone.0261998.s002.docx]

**S2 Appendix. Multilevel Model on Missing Neighbourhood Characteristics for Ageing in Place and Physical Activity**

Level 1:

$$Y_{ij}= \beta0j+ {\beta1}_{j} {age\_centred}_{ij}+{\beta2}_{j} {gender}_{ij}+ {\beta3}_{j} {marital status}_{ij}+\beta4_{j} {education}_{ij}+ {\beta5}_{j} {income}_{ij}+ {\beta6}_{j}{number of chronic diseases}_{ij}+ {\beta7}_{j} {overall missing neighbourhood characteristics}_{ij}+ e_{ij}$$

Level 2:

$$\beta0j= \gamma_{00}+u_{0j}$$

${\beta1}_{j}$ = $\gamma_{10}$

${\beta2}_{j}$ = $\gamma_{20}$

${\beta3}_{j}$ = $\gamma_{30}$

$$\beta4j= \gamma_{40}+u_{4j}$$

${\beta5}_{j}$ = $\gamma_{50}$

${\beta6}_{j}$ = $\gamma_{60}$

${\beta7}_{j}$ = $\gamma_{70}$

| **Model** | **- 2 log likelihood** | **AIC*** |
| --- | --- | --- |
| With no covariates and no random intercept | 2880 | 2884 |
| With no covariates and a random intercept | 2875 | 2881 |
| With all covariates and a random intercept | 2264 | 2284 |
| With all covariates, a random intercept and a random slope for education | 2255 | 2271 |

*Akaike information criterion
