# Supplementary material for: Age-friendly neighbourhoods and physical activity of older Surinamese individuals in Rotterdam, the Netherlands
Source: PLoS One. 2022 Jan 27;17(1):e0261998. doi: 10.1371/journal.pone.0261998 (PMC8794150; doi:10.1371/journal.pone.0261998)
Supplement: S3 Appendix — (DOCX) [file pone.0261998.s003.docx]

**S3 Appendix. Physical Activity Before and After Implementation of COVID-19 Measures***

|  | Before COVID-19 measures (n=215) | After COVID-19 measures (n=376) | *p* |
| --- | --- | --- | --- |
|  | Mean (SD) | Mean (SD) |  |
| Physical activity | 3.66 (2.32) | 3.79 (2.37) | 0.51 |

*One way paired t-test was performed in order to compare physical activity among participants who filled in the questionnaire before and after the implementation of COVID-19 measures in the Netherlands. SD, standard deviation.
