# Supplementary material for: Age-friendly neighbourhoods and physical activity of older Surinamese individuals in Rotterdam, the Netherlands
Source: PLoS One. 2022 Jan 27;17(1):e0261998. doi: 10.1371/journal.pone.0261998 (PMC8794150; doi:10.1371/journal.pone.0261998)
Supplement: S4 Appendix — (DOCX) [file pone.0261998.s004.docx]

**S4 Appendix. COVID-19 Measures Taken in the Netherlands** [1]

March 12, 2020

- Gatherings of more than 100 people are to be cancelled throughout the Netherlands.
- People throughout the Netherlands are encouraged to work from home or stagger their working times if possible.
- For everyone in the Netherlands: stay at home if you have a cold, a cough, a sore throat or a fever. Avoid contact with others (social distancing). Only call your doctor if your symptoms worsen.
- For vulnerable individuals (older individuals and individuals with low resistance): avoid large groups and public transport. In general, people are urged to limit visits to vulnerable individuals.
- Universities and institutions of higher professional education (HBO) are requested to offer online lectures instead of large-scale lectures.
- Primary schools, secondary schools, secondary vocational schools (MBO) and childcare centers will remain open as usual.

March 16, 2020

Additional measures:

- Restaurants, bars and ‘contact professions’ (e.g. hairdressers, nail salons and beauticians) are closed.
- Sports and fitness clubs are closed.
- Primary schools, secondary schools, secondary vocational schools (MBO) and childcare centers will be closed.
- Keep 1.5m distance of individuals outside the household.

March 19, 2020

- Nursing homes and small-scale housing in elderly care will be closed for visitors and others who are not necessary for basic care.

March 23, 2020

Additional measures:

- A maximum of 3 people are allowed to visit.
- A maximum of 3 people are allowed to be together outside, with 1.5m distance.
- The final exams of secondary school students are canceled this year.
- All gatherings and events are canceled until June 1^st^. Religious gathering’s, funerals and weddings are allowed, provided that less than 30 individuals are present and 1.5m distance can be taken into account.

6 April, 2020

Lessening of the measures:

- Sports and fitness clubs will be open again,
- Primary schools, secondary schools, secondary vocational schools (MBO) and childcare centers will be open again.

April 29, 2020

Lessening of the measures:

- Children (< 13 years) are allowed to sport together under supervision. Matches are not allowed.
- Young people (13 – 18 years) are allowed to sport together under supervision with 1.5m distance. Matches are not allowed.
- Elite athletes are allowed to resume indoor and outdoor training, with 1.5m distance. Matches are not allowed.

May 11, 2020

Additional measures:

- Primary schools and childcare centers will be open again.
- Primary schools will halve the group size; children will go half of the time to school, the other half they will be educated at home.
- Libraries will be open again.

May 19, 2020

Additional measures:

- For cinemas, restaurants and cafes, and cultural institutions (such as concert halls and theaters), the maximum of 30 people applies excluding staff and artists.
- Wearing a mouth mask is mandatory in public transport
- Sport clubs, casino’s, sauna’s and sex services will open on July 1^st^.

June 24, 2020

Additional measures:

- Inside: more than 100 individuals are allowed, if you reserve a place. A health check will be available (e.g. for cinema’s, restaurants, weddings and funerals).
- Outside : more than 250 individuals are allowed with a permanent place. You have to reserve and there will be a health check.

1. **New measures to stop spread of coronavirus in the Netherlands** [<https://www.government.nl/latest/news/2020/03/12/new-measures-to-stop-spread-of-coronavirus-in-the-netherlands>]
