# Supplementary material for: Age-friendly neighbourhoods and physical activity of older Surinamese individuals in Rotterdam, the Netherlands
Source: PLoS One. 2022 Jan 27;17(1):e0261998. doi: 10.1371/journal.pone.0261998 (PMC8794150; doi:10.1371/journal.pone.0261998)
Supplement: S5 Appendix — (DOCX) [file pone.0261998.s005.docx]

**S5 Appendix. Physical Activity of Creole, Hindustani and Other Surinamese***

|  | Surinamese Creole (n=259) | Surinamese Hindustani (234) | Other (n=127) | *p* |
| --- | --- | --- | --- | --- |
|  | Mean (SD) | Mean (SD) | Mean (SD) |  |
| Physical activity | 3.70 (2.24) | 3.65 (2.45) | 3.87 (2.34) | 0.68 |

* Analysis of Variance (ANOVA) was performed in order to compare physical activity among participants based on their ethnicity. SD, standard deviation.
